# Efficacy and Safety of Topical Compound Heparin Sodium Allantoin Gel (Main Components: Onion Extract Quercetin) for the Treatment of Rosacea

**DOI:** 10.1111/jocd.70129

**Published:** 2025-04-03

**Authors:** Zining Xu, Biao Yu, Yuxin Qing, Shuhong Ye, Bingyang Xu, Yuanqin Wang, Bin Zhao, Hong Sun, Na Wu, Jiawen Wu

**Affiliations:** ^1^ Department of Dermatology The Second Affiliated Hospital of Xi'an Jiaotong University Xi'an China; ^2^ Department of Dermatology Taihe Hospital, Hubei University of Medicine Shiyan China; ^3^ Department of Dermatology Xi'an International Medical Center Hospital Xi'an China; ^4^ Department of Neurology The Second Affiliated Hospital of Xi'an Jiaotong University Xi'an China; ^5^ Department of Nursing The Medicine of Xi'an Jiaotong University Xi'an China

**Keywords:** gel formulations, papulopustular rosacea, skin barrier function, skin hydration, trans‐epidermal water loss

## Abstract

**Background:**

The management of papulopustular rosacea presents a significant clinical challenge. Anti‐inflammatory and vasoconstrictive treatments are ineffective in the rapid amelioration of the dryness, burning, and itching caused by skin barrier damage in patients with papulopustular rosacea.

**Aims:**

To assess the efficacy and safety of the topical application of compound heparin sodium allantoin gel to treat rosacea.

**Methods:**

Eighty‐two patients participated in this randomized, prospective, single‐center, and controlled trial. The Clinician Erythema Assessment score, Investigator Global Assessment score, transepidermal water loss, and skin hydration were evaluated at 0, 2, 4, 8, and 12 weeks. Rosacea‐specific quality of life score, itching, dryness, burning, Global Aesthetic Improvement Scale, and Patient Self‐Assessment grades were also assessed.

**Results:**

Compared with the traditional therapy group, the 8‐week and 12‐week topical application of compound heparin sodium allantoin gel to treat rosacea significantly decreased Clinician Erythema Assessment/Investigator Global Assessment grades, burning and itching grades, and rosacea‐specific quality of life scores. Compound heparin sodium allantoin gel significantly improved the skin barrier with hydration and significantly decreased trans‐epidermal water loss. For patients with Demodex infestation, externally applied compound heparin sodium allantoin gel was associated with better rosacea treatment outcomes and improved skin barrier function than externally applied hyaluronic acid. This may be attributable to the inhibition of abnormal demodex, improved skin barrier, and repair of minor skin wounds.

**Conclusions:**

Compound heparin sodium allantoin gel effectively improved facial erythema, alleviated ithching and burning sensations, and improved patients' quality of life.

**Trail Registration:**

ClinicalTrials.gov identifier: ChiCTR2400087948

## 1 Introduction

Patients with rosacea often feel flushing and itching on the face and based on disease progression, the clinical typology can be divided into erythemato‐telangiectatic, papulopustular, phymatous, and ocular rosacea. The incidence of papulopustular rosacea (PPR) is the highest, at approximately 1.2%. Abnormal proliferation of demodex on the face is an important initiating and exacerbating factor of rosacea; the combination of minor wounds formed by scratching and abnormal proliferation of demodex is a reason it is difficult to improve clinical symptoms and quality of life for patients with PPR. Currently, PPR management is a clinical challenge. Anti‐inflammatory and vasoconstrictive treatments do not rapidly and effectively ameliorate the stinging, burning, and itching caused by skin barrier damage in these patients [1, 2, 3, 4]. The Food and Drug Administration has not approved drugs for the treatment of rosacea that can enhance the skin barrier function [5]. Studies indicate that hyaluronic acid (HA: Hyaluronic acid repair biological excipient, Hainan Zhongkangyue Medical Instrument Co. Ltd., Haikou City, Hainan Province, China) alleviates symptoms such as dryness, burning, and itching; however, its single‐component formulation and variability among preparations limit its efficacy [6]. Therefore, it is particularly important to improve the quality of life of patients with PPR by rapidly and effectively enhancing the skin barrier function, promoting anti‐inflammatory and anti‐angiogenic effects, and inhibiting vascular endothelial cell proliferation.

Skin barrier damage experienced by patients with rosacea is similar to that associated with atopic dermatitis, including decreased skin hydration and increased trans‐epidermal water loss(TEWL) [7]. Additionally, studies have shown that the rosacea pathogenesis is related to genetic and natural immune abnormalities and neurovascular dysfunction [8]. Compound heparin sodium allantoin gel (CHSAG: Contractubex. Merz Pharmaceuticals GmbH, D‐60318 Frankfurt/Main, Germany) is a mixture of onion extract containing quercetin (as the main component), heparin sodium, and allantoin [9]. CHSAG promotes anti‐inflammatory effects, inhibits infection, angiogenesis, vascular endothelial hyperplasia, and smooth muscle cell proliferation, stabilizes epidermal microorganisms, and repairs the skin barrier [10]. Therefore, this study evaluated the efficacy and safety of externally used CHSAG for the treatment of PPR.

## 2 Materials and Methods

### 2.1 Study Design and Procedure

This randomized, prospective, single‐center, controlled study was conducted at Xi'an Jiaotong University in Xi'an, Shaanxi Province, China, from March to November 2023. The study was approved (approval number: 2023254), by the Institutional Review Board of the Second Affiliated Hospital of Xi'an Jiaotong University. Informed consent was obtained from all participants.

The inclusion criteria were aged 18–60 years; fulfilled the 2021 Chinese clinical guidelines for the diagnosis and treatment of rosacea; and absence of liver and kidney function abnormalities, metabolic abnormalities, and bone system diseases. The exclusion criteria were current pregnancy, currently breastfeeding, desire to conceive within 6 months, allergy to CHSAG, inability to take doxycycline, malignant tumors, psychosocial disorders, and other serious systemic diseases.

We used a computer‐based random number generator to randomly divide a total of 82 patients into two groups of 41 patients each: experimental group (EG) and control group (CG). Patients with PPR were randomly numbered and administered treatment according to their group. The EG group received oral doxycycline 50 mg once daily and CHSAG (Composition: 100 g gel contains: 10.0 g Extr. cepae, 5000 IU heparin sodium, 1.0 g allantoin) twice daily for 8 weeks, 1–2 g/dose, whereas the CG group received oral doxycycline 50 mg once daily and HA emulsion (Composition: Sodium hyaluronate, purified water and glycerin, sodium benzoate, 2‐phenoxyethanol) twice daily for 8 weeks, 1–2 g/dose.

At 0–8 weeks, the infection status associated with Demodex mites was identified using fluorescence microscopy (a positive result defined as the presence of ≥ 5 mites per cm^2^ of skin, based on established criteria for pathogenic Demodex counts) [11], and patients' basic information was recorded [12]. At 0, 2, 4, 8, and 12 weeks, the Clinical Erythema Assessment (CEA) score, inflammatory lesion count, Investigator Global Assessment (IGA) score, TEWL, and skin hydration (Test instrument: DermaLab Combo Multiparameter Skin Analysis System [Cortex Technology, Hadsund, Denmark]. Test conditions: Ambient relative humidity 25%–55%, temperature 20°C‐25°C) were evaluated [13]. At 0 and 8 weeks, rosacea‐specific quality of life, itching, dryness, burning sensation, and global aesthetic scores were assessed, and clinical photographs were obtained using the eight‐spectrum facial skin detector. Rosacea‐specific quality of life and Patient Self‐Assessment (PSA) scores were re‐evaluated after 12 weeks, and patient satisfaction scores were calculated. The main evaluation index was the CEA score, and the main outcome was improvement in the CEA score of at least 1 point at 8 weeks [14]. The criterion for a superior outcome in the EG was an improvement rate > 10% compared with that of the CG. The scoring scale was completed using a questionnaire. The participants were not allowed to use any other drugs or undergo physical therapy or surgery that might affect rosacea during the study.

### 2.2 Statistical Analysis

The sample size for the statistical analysis was calculated by assuming that the effective rates for the CG and EG were 25% and 62.5%, respectively, and that the withdrawal rate was 20% [15]. A population size of 35 patients per group was estimated to provide at least 85% power to detect statistically significant differences in the primary endpoint (two‐sided significance level, 0.05). The *t*‐test, Mann–Whitney *U* test, Fisher's exact test, Chi‐square test, and Spearman's rank correlation coefficient were conducted based on the data type. SPSS version 18.0 (SPSS Inc., Chicago, IL, USA) was used to analyze the data. Patients with missing data were excluded.

## 3 Results

### 3.1 Baseline Patient Data

**FIGURE 1:**
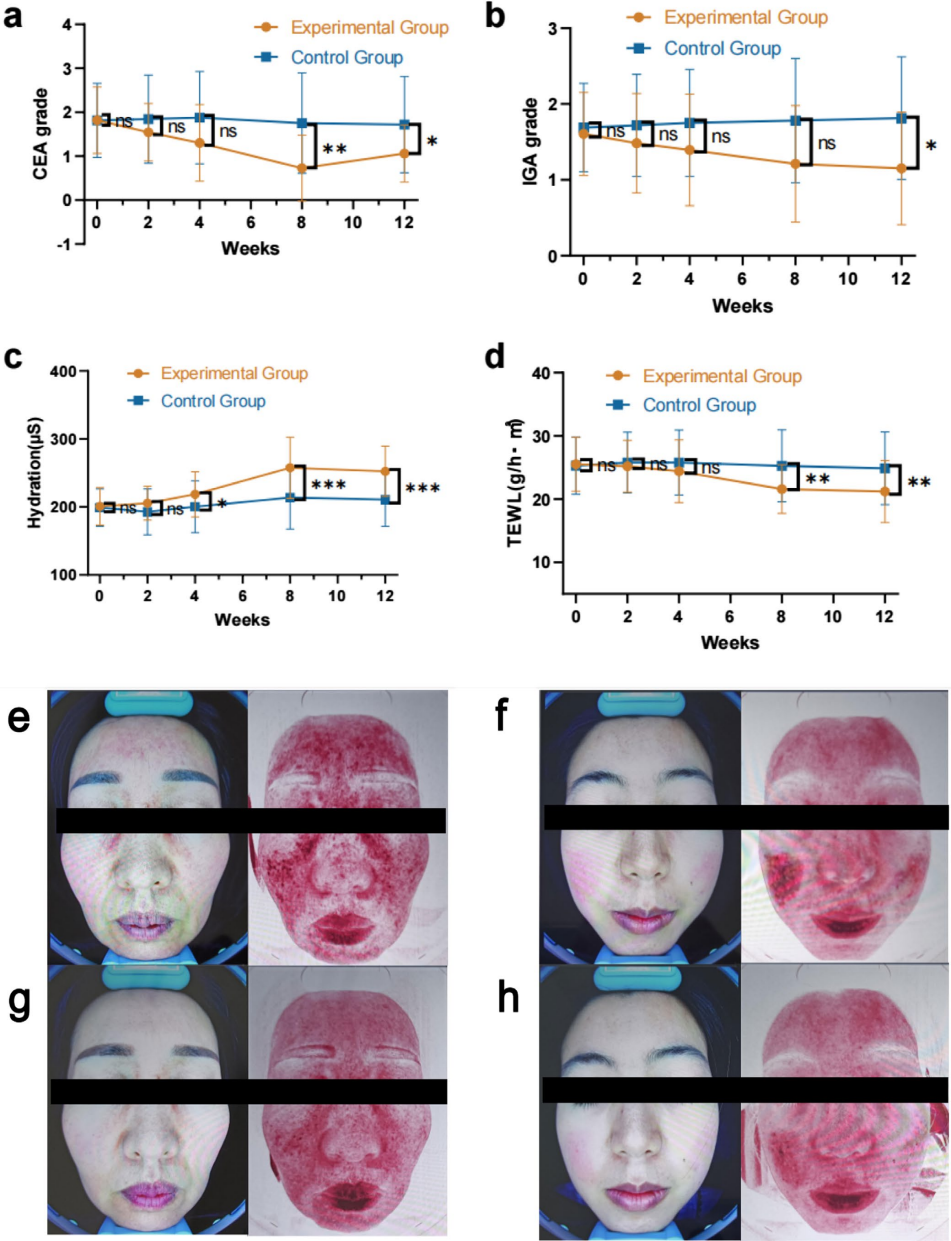
Compared with the control group, significant improvements are observed in the experimental group in Clinical Erythema Assessment and Investigator Global Assessment scores, hydration, and transepidermal water loss. The mean changes in the Clinical Erythema Assessment (CEA) and Investigator Global Assessment (IGA) scores (a, b) and hydration and transepidermal water loss (TEWL) (c, d) are compared with those at baseline. Representative images of patients with rosacea after the external use of compound heparin sodium allantoin gel (CHSAG) or hyaluronic acid treatment at baseline and 8 weeks (e, f, g, h) ***p < 0.001. The error bars indicate the standard error of the mean.

A total of 96 patients participated in the trial; however, 14 withdrew before randomization. The remaining 82 patients were randomly divided into the EG (*n* = 41) and CG (*n* = 41). Sixty‐five patients (EG, *n* = 33; CG, *n* = 32) completed follow‐up. At baseline, the two groups were almost balanced regarding demographic (Table 1) and scale characteristics (Table 2). In the EG group, the means of the BMI scores and Rosacea Quality of Life scores relative to the overall standard deviation were greater than in the CG group (Cohen's d = 0.212, Cohen's d = 0.385). The average age of patients in these groups was 29.39 years and 28.27 years (range: 18–60 years), respectively. All enrolled patients were from northwest China and had a median history of rosacea of 3 years; furthermore, most of the patients were female (EG, 90.24%; CG, 87.80%) (Table 1). According to the CEA or IGA, most patients had mild to moderate erythema (Table 1; Figure 1).

**TABLE 1:** Demographic characteristics.

Characteristics	EG (*n* = 41)	CG (*n* = 41)	*p* (CI = 95%)	Cohen's d effect size (CI = 95%)
Sex, No. (%)				
Men	4 (9.76)	5 (12.20)	0.729	
Women	37 (90.24)	32 (87.80)		
Age in years, median (IQR)	29.39 (23,32.5)	28.27 (23.5,33)	0.54	0.156
Height, mean (SD), cm	164.00 (6.99)	164.12 (8.33)	0.943	−0.016
Weight, mean (SD), kg	58.59 (9.38)	57.32 (8.48)	0.523	0.014
Rosacea history in years, median (IQR, mode)	3.93 (2,5,3)	4.17 (2.5, 4.25, 3)	0.298	—
Family history (%)	16 (39.02)	21 (51.22)	0.267	—
BMI, mean (SD)	21.74 (2.89)	21.21 (2.08)	0.340	0.212

Abbreviations: BMI, body mass index; CG, control group; CI, confidence interval; EG, experimental group; IQR, interquartile range; No, number; SD, standard deviation.

**TABLE 2:** Scale characteristics.

Characteristics	EG (*n* = 41)	CG (*n* = 41)	*p* (CI = 95%)	Cohen's d effect size (CI = 95%)
Baseline RQol, mean (SD), score	56.22 (16.58)	52.05 (13.63)	0.217	0.385
Baseline SCH, mean (SD), μS	199.60 (29.86)	199.51 (28.11)	0.989	0.065
Baseline TEWL, mean (SD), g/h·m^2^	25.33 (4.75)	25.19 (4.22)	0.890	0.050
Demodex infection (%)	18 (43.90)	20 (48.78)	0.658	—
CEA, No. (%)				
Almost clear = 1	15 (36.59)	17 (41.46)	0.637	—
Mild = 2	19 (46.34)	15 (36.59)		
Moderate = 3	5 (12.20)	8 (19.51)		
Severe = 4	2 (4.88)	1 (2.44)		
IGA, No. (%)				
Almost clear	19 (46.34)	16 (39.02)	0.775	—
Mild	20 (48.78)	22 (53.66)		
Moderate	2 (4.88)	3 (7.32)		
Severe	0 (0)	0 (0)		
PSA, No. (%)				
Almost clear	8 (22.86)	9 (21.95)	0.827	—
Mild	22 (53.66)	21 (51.22)		
Moderate	7 (17.07)	9 (21.95)		
Severe	4 (9.76)	2 (4.88)		
Itching sensation				
Almost clear = 0	10 (24.39)	15 (36.59)	0.161	—
Mild = 1	23 (56.10)	14 (34.15)		
Moderate = 2	6 (16.63)	11 (26.83)		
Severe = 3	2 (4.88)	1 (2.44)		
Dryness sensation				
Almost clear = 0	2 (4.88)	5 (12.20)	0.194	—
Mild = 1	18 (43.90)	24 (58.54)		
Moderate = 2	17 (41.46)	9 (21.95)		
Severe = 3	4 (9.76)	3 (7.32)		
Burning sensation				
Almost clear = 0	17 (41.46)	15 (36.59)	0.959	—
Mild = 1	15 (36.59)	16 (39.02)		
Moderate = 2	8 (22.86)	9 (21.95)		
Severe = 3	1 (2.86)	1 (2.86)		

Abbreviations: CEA, clinical erythema assessment; CG, control group; CI, confidence interval; EG, experimental group; IGA, investigator global assessment; No, number; PSA, patient self‐assessment; RQol, rosacea‐specific quality of life; SCH, Stratum Corneum Hydration; SD, standard deviation; TEWL, transepidermal water loss.

### 3.2 Improvements in CEA, IGA, and Skin Barrier Function Scores of the EG Were Significantly Greater Than Those of the CG

Compared with the CG, the EG had greater and faster decreases in both CEA and IGA scores (Figure 1a,b and Figure S1a). At 2, 4, and 8 weeks, no significant differences in CEA and IGA scores were observed between these groups. At 8 and 12 weeks, with a superior limit of 10%, the proportion of patients with a decrease of at least 1 point in the CEA score was higher in the EG (24/33 [72.73%]; 20/33 [60.61%]) than in the CG (8/32 [25.00%]; 8/32 [25.00%]); these results were significant (95% confidence interval [CI], *p* = 0.002, odds ratios [OR] = 8.000; 95% CI, *p* = 0.037, OR = 4.615). At 8 and 12 weeks, the proportion of patients with an improvement of at least 1 point in the IGA score was higher in the EG (15/33 [45.45%]; 16/33 [48.48%]) than in the CG (5/32 [15.63%]; 4/32 [12.50%]); these results were statistically significant at 12 weeks (95% CI, *p* = 0.023, OR = 6.588) but not at 8 weeks (95% CI, *p* = 0.083, OR = 4.500). The difference in the hydration scores of the EG and CG was statistically significant from 4 weeks, with the *p*‐value gradually decreasing with increasing follow‐up time (95% CI, *p* = 0.095, Cohen's d = 0.420 at 2 weeks; 95% CI, *p* = 0.046, Cohen's d = 0.510 at 4 weeks; 95% CI, *p* < 0.001, Cohen's d = 0.941 at 8 weeks; 95% CI, *p* < 0.001, Cohen's d = 1.069 at 12 weeks) (Figure 1c,d and Figure S1b). No significant difference in the change in transepidermal water loss (TEWL) was observed between the EG and CG during the first 2 and 4 weeks; however, a significant difference was observed at 8 and 12 weeks (95% CI, *p* = 0.004, Cohen's d = −0.744; 95% CI, *p* = 0.008, Cohen's d = −0.677) (Figure 1c,d and Figure S1b). Representative images of patients with rosacea from EG and CG groups at baseline and 8 weeks are shown (Figure 1e–h, Figures S1c, S1d, S1e, and S1f).

### 3.3 EG Experienced Significantly Improved Itching, Dryness, and Burning Sensations, Global Aesthetic Improvement Scale, PSA, and Rosacea‐Specific Quality of Life Scores

**FIGURE 2:**
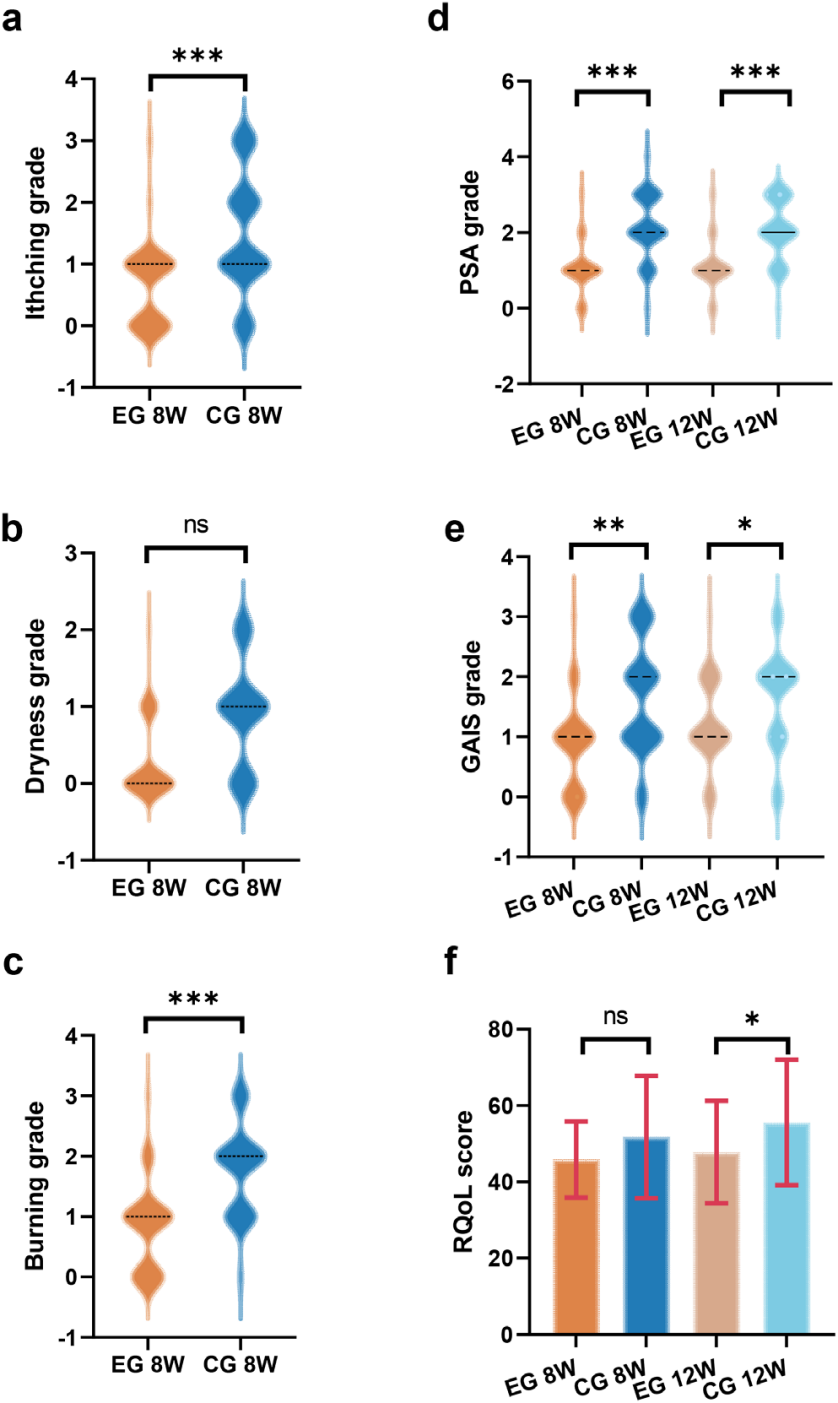
Comparison of clinical symptom scores between the experimental group (EG) and control group (CG) at 8 and 12 weeks. Violin plots and bar charts illustrate the distribution of (a) itching, (b) dryness, and (c) burning grades at 8 weeks, and (d) PSA, (e) GAIS, and (f) RQoL scores at 8 and 12 weeks. The EG demonstrated significant reductions in itching and burning grades compared to the CG at 8 weeks (***p < 0.001). GAIS and PSA scores improved significantly at 12 weeks in the EG (*p < 0.05, **p < 0 0.01, ***p < 0.001, “ns” indicates no significant difference).

At 8 weeks, the EG experienced a significant decrease in itching and burning sensation compared with the CG (95% CI, *p* < 0.001; 95% CI, *p* < 0.001) (Figure 2a,c). Dryness was not significantly different between the two groups (95% CI, *p* = 0.086) (Figure 2b). Compared with the CG, the EG experienced significant differences in Global Aesthetic Improvement Scale and PSA scores at 8 and 12 weeks, with the difference in the PSA score being more significant (PSA score: 95% CI, *p* < 0.001; 95% CI, *p* < 0.001. Global Aesthetic Improvement Scale score: 95% CI, *p* = 0.007; 95% CI, *p* = 0.033) (Figure 2d,e). The difference in rosacea‐specific quality of life was not statistically significant at 8 weeks (95% CI, *p* = 0.077, Cohen's d = −0.446), whereas it was significant at 12 weeks (95% CI, *p* = 0.040, Cohen's d = −0.520) (Figure 2f and Figure S1b).

### 3.4 Correlation Between Improvement in Skin Barrier Function and the CEA Score With Externally Used CHSAG

**FIGURE 3:**
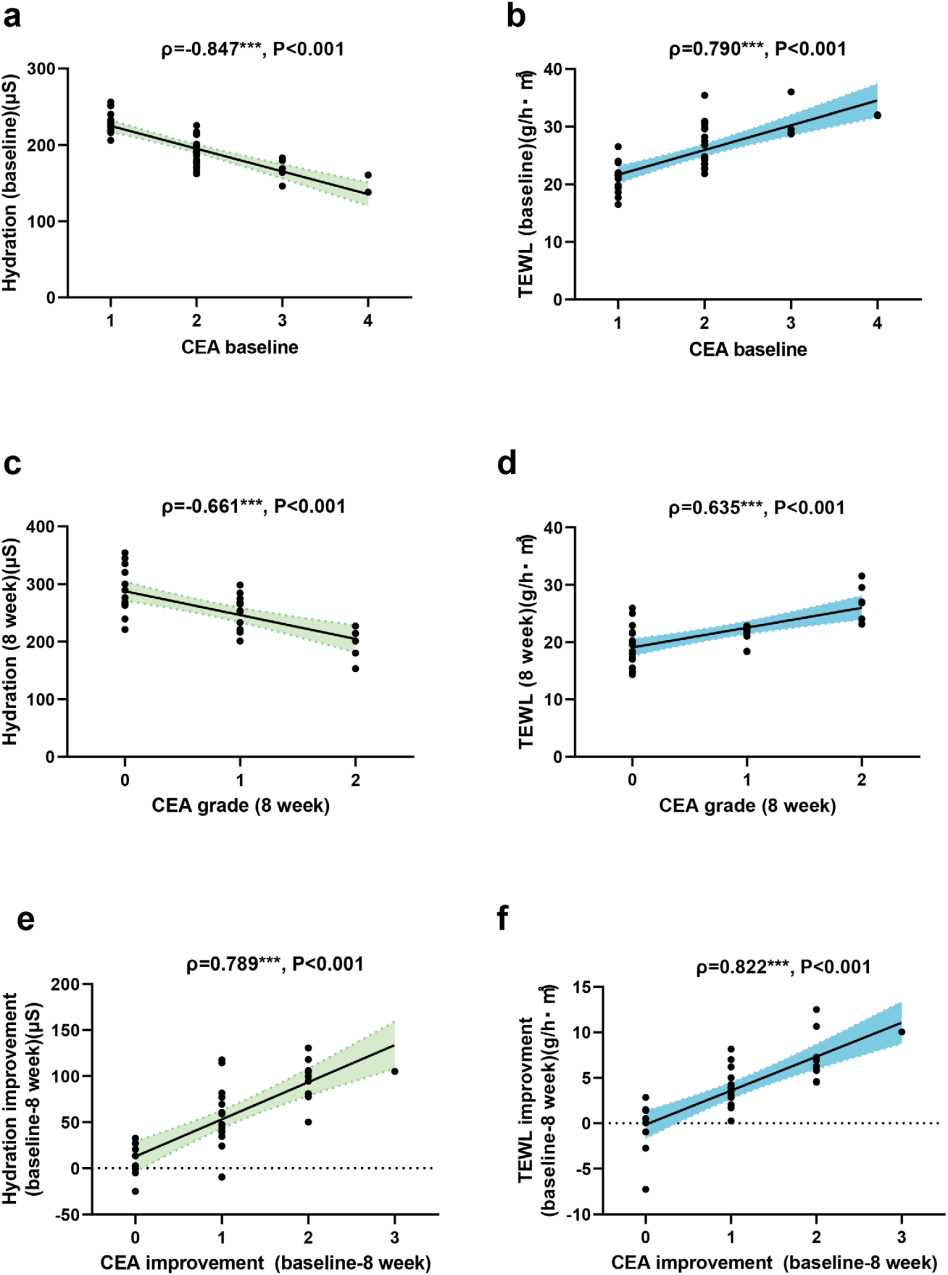
Correlation between clinical erythema assessment grades and skin barrier parameters. Correlations of skin hydration and transepidermal water loss (TEWL) with the CEA grades at baseline (a, b) and 8 weeks (c, d) in the experimental group (EG). Correlations of skin hydration and TEWL improvements with the CEA grades of the EG at 8 weeks (e, f) (***p < 0.001, the error bars indicate the standard error of the mean).

We evaluated skin hydration and TEWL to determine the effects of CHSAG on the skin barrier. We found a correlation among skin hydration, TEWL, and the CEA score. Our data showed that skin hydration and TEWL were correlated with the CEA score at baseline (*ρ* = −0.847, *p* < 0.001; *ρ* = 0.790, *p* < 0.001) (Figure 3a,b) and 8 weeks (*ρ* = −0.661, *p* < 0.001; *ρ* = 0.635, *p* < 0.001) (Figure 3c,d). An additional analysis showed that the improvement in skin hydration and TEWL during CHSAG treatment was closely related to the improvement in the CEA score (*ρ* = 0.789, *p* < 0.001; *ρ* = 0.822, *p* < 0.001) (Figure 3e,f).

### 3.5 CHSAG Had a Better Effect on Patients With Demodex Infections

**FIGURE 4:**
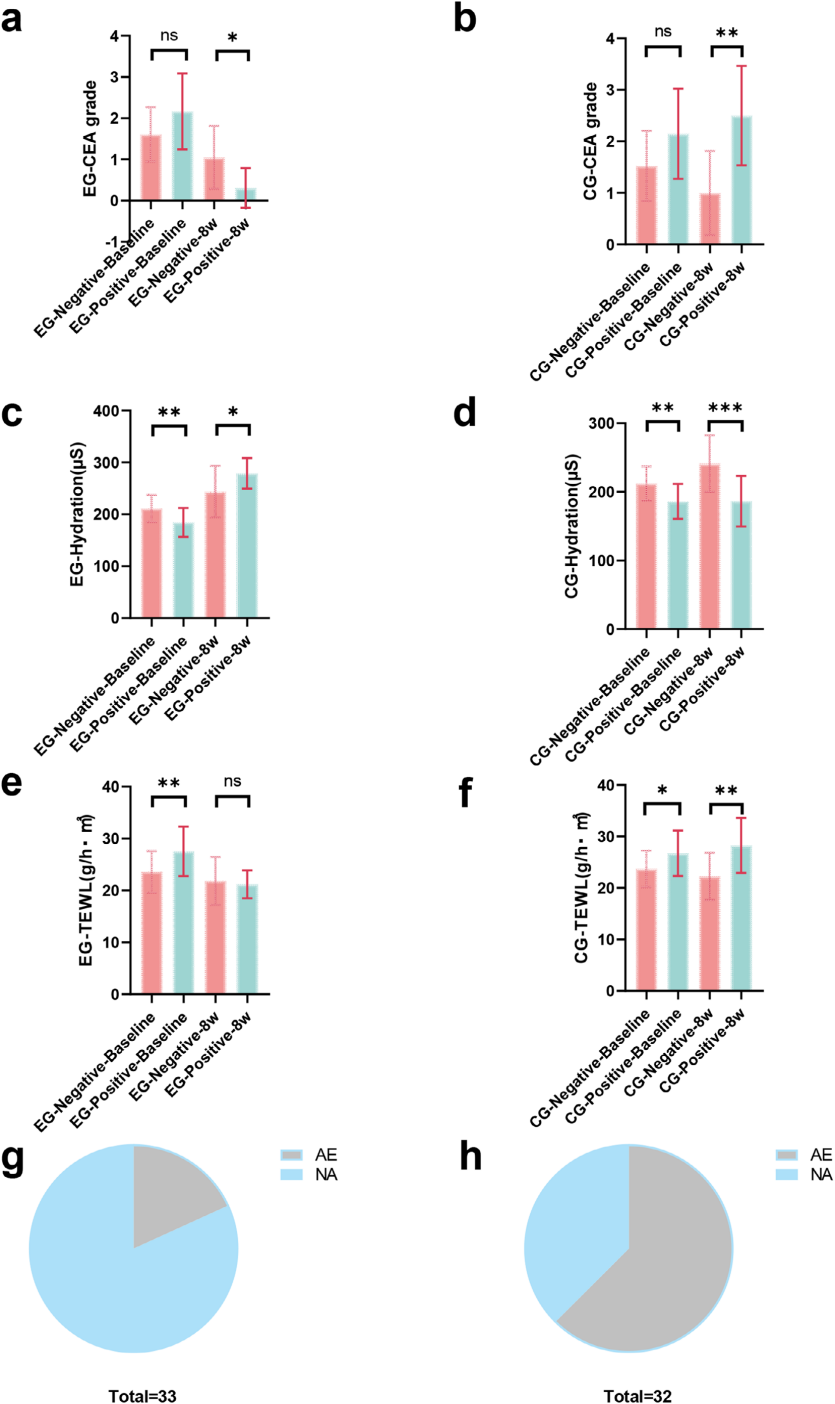
Skin hydration and transepidermal water loss are significantly different between the subgroups. The mean clinical erythema assessment grades are not different between the negative and positive Demodex infection subgroups of the experimental group and control group at baseline and 8 weeks (a, b). The skin barrier of the negative Demodex infection subgroups is better than that of the positive subgroups at baseline. At 8 weeks, the skin barrier of the positive Demodex infection subgroups of the experimental group (EG) exhibited better improvement than that in the negative Demodex infection subgroups; however, this effect was not observed in the control group (CG) (c–f). Adverse events (AEs) experienced by patients in the EG (g) and CG (h). NA, no adverse events. ***p < 0.001, **p < 0.05, and *p < 0.01. The error bars indicate the standard error of the mean.

To perform a subgroup analysis, we divided the patients in the EG and CG into negative and positive subgroups according to their Demodex infection status. We found no significant difference in the CEA score between the negative and positive subgroups of the EG and CG at baseline (95% CI, *p* = 0.180; 95% CI, *p* = 0.106) (Figure 4a,b). At 8 weeks, the CEA score of the positive subgroup of the EG was significantly lower than that of the negative subgroup; however, the CEA score of the positive subgroup of the CG was significantly higher than that of the negative subgroup (95% CI, *p* = 0.017; 95% CI, *p* = 0.004) (Figure 4a,b). A significant difference in skin hydration was observed between the negative and positive subgroups of the EG and CG at baseline; skin hydration in the positive subgroup was lower than that in the negative subgroup (95% CI, *p* = 0.003; 95% CI, *p* = 0.106) (Figure 4c,d). At 8 weeks, skin hydration in the positive subgroup of the EG was significantly higher than that in the negative subgroup (95% CI, *p* = 0.002) (Figure 4c), whereas skin hydration in the positive subgroup of the CG was significantly lower than that in the negative subgroup (95% CI, *p* < 0.001) (Figure 4d). A significant difference in TEWL was observed in the negative and positive subgroups of the EG and CG at baseline; TEWL in the positive subgroup was higher than that in the negative subgroup (95% CI, *p* = 0.007; 95% CI, *p* = 0.016) (Figure 4e,f). At 8 weeks, no significant difference in TEWL was observed between the positive and negative subgroups of the EG (95% CI, *p* = 0.631) (Figure 4e), whereas TEWL in the positive subgroup of the CG was significantly higher than that in the negative subgroup of the CG (95% CI, *p* = 0.002) (Figure 4f).

### 3.6 Patients Treated With CHSAG Did Not Experience Obvious Side Effects

Adverse reactions were defined as flushing and transient skin tingling, worsening of itching, burning, and dryness sensation during rosacea treatment. During the study period, 65 patients reported 26 adverse events (6 in the EG and 20 in the CG). The proportion of patients in the CG who experienced adverse events was higher than that in the EG (EG: 6/33 [18.18%]; CG: 20/32 [62.50%]; 95% CI, *p* < 0.001, Cohen's d = 0.133) (Figure 4g,h and Figure S1a). Notably, adverse reactions including itching and burning in the six patients in the EG occurred 2 weeks before the initial application of CHSAG. No adverse reactions occurred after 2 weeks (Table 3). In the CG, 14 patients experienced worsening of itching and burning sensations, 5 patients felt flushing and transient skin stinging, and 1 patient felt more dryness at the facial lesion sites (Table 3). No serious adverse events or deaths were observed during this study, and none of the patients discontinued follow‐up because of adverse reactions.

**TABLE 3:** Adverse reactions.

Time	EG (*n* = 6%)	CG (*n* = 20%)	*p* (CI = 95%)	OR
Itching, No.	2 weeks (*n* = 4), Total (*n* = 4)	2 weeks (*n* = 2), 4 weeks (*n* = 1), 6 weeks (*n* = 2), 8 weeks (*n* = 1), 12 weeks (*n* = 1), Total (*n* = 7)	0.518	0.433
Burning, No.	2 weeks (*n* = 2), Total (*n* = 26.06)	2 weeks (*n* = 3), 4 weeks (*n* = 2), 6 weeks (*n* = 2), 8 weeks (*n* = 0), 12 weeks (*n* = 0), Total (*n* = 7)	0.158	0.230
Stinging, No.	Total (*n* = 0)	2 weeks (*n* = 3), 4 weeks (*n* = 1), 6 weeks (*n* = 1), 8 weeks (*n* = 0), 12 weeks (*n* = 0), Total (*n* = 5)	0.056	—
Dryness, No.	Total (*n* = 0)	2 weeks (*n* = 0), 4 weeks (*n* = 1), 6 weeks (*n* = 0), 8 weeks (*n* = 0), 12 weeks (*n* = 0), Total (*n* = 1)	1.000	—

Abbreviations: CG, control group; CI, confidence interval; EG, experimental group; OR, odds ratios.

## 4 Discussion

This study demonstrated that patients treated with CHSAG exhibited significantly greater improvements in CEA, IGA, PSA, the Global Aesthetic Improvement Scale, skin barrier function, and rosacea‐specific quality of life scores compared to those treated with HA. Additionally, CHSAG treatment was associated with significant reductions in itching, improved skin hydration, and alleviation of burning sensations. Notably, CHSAG was particularly effective in patients with Demodex infections. These findings suggest that CHSAG effectively reduces facial erythema, mitigates skin dryness and burning sensations, and enhances the overall quality of life in individuals with rosacea.

The inflammatory pathways are currently associated with rosacea pathogenesis include immune (congenital and adaptive), neurocutaneous dysregulation, and angiogenesis. Innate immune activation leads to the upregulation of keratinocyte‐derived toll‐like receptor 2 (TLR2) and protease‐activated receptor 2 (PAR2), along with an increased infiltration of inflammatory cells, including neutrophils, macrophages, and mast cells [8, 16]. The adaptive immune response is driven by the activation of type 1 T‐helper (Th1) and type 17 T‐helper (Th17) lymphocytes and their corresponding immune mediators, which amplify inflammation and promote further immune activation, with an associated increase in CD4+ T cells [8, 16]. Dysregulation of the neurocutaneous mechanism is manifested by the activation of transient receptor potential vanilloid 1 (TRPV1), which further stimulates the release of vasoactive peptides from neuronal fibers and histamine and serotonin from mast cells [9]. CHSAG contains a combination of onion extract (with quercetin as the primary active component), heparin sodium, and allantoin [10]. Recent studies have highlighted the potent anti‐inflammatory and immunoregulatory properties of quercetin, demonstrating its ability to downregulate TLR2 and PAR2 [17], modulate the balance of Th1, T‐helper 2 (Th2), and Th17 cells [18], attenuate CD4^+^ T‐cell and neutrophil infiltration, and suppress the transcription of chemokines in vivo [19]. Additionally, quercetin has been shown to mitigate TRPV1 activation and reduce the production of pro‐inflammatory cytokines in macrophages and mast cells [20, 21]. Beyond its immunomodulatory effects, quercetin exhibits anti‐inflammatory, antioxidant, moisturizing, and anti‐melanogenic properties in human keratinocytes. Notably, it significantly inhibits pro‐inflammatory cytokines and chemokines in HaCaT cells. Furthermore, quercetin downregulates inflammation‐associated transcripts in human dermal microvascular endothelial cells and reduces cluster of differentiation 31(CD31)‐positive vascular structures in the skin lesions of rosacea‐like mice [19]. Importantly, quercetin activates activator protein‐1 (AP‐1) signaling pathways, which may play a role in preventing postinflammatory hyperpigmentation associated with rosacea [22]. In addition to quercetin, the topical application of heparin sodium has been reported to prevent infection and enhance skin microcirculation [23], while allantoin contributes to improved local circulation and alleviates congestion and edema [24]. The results of the current study showed a positive effect of a topical application of CHSAG on the treatment of PPR; however, the specific mechanism still needs to be further verified.

A change in the skin barrier is an important clinical symptom associated with the occurrence and aggravation of rosacea [25]. Keratin 14, keratin 17, filaggrin, and loricrin are the main components of healthy skin [26]. An increase in serine protease activity and upregulation of antimicrobial peptides play important roles in the pathogenesis of rosacea [27]. Quercetin may increase the expression levels of filaggrin and transglutaminase 1 through the NF‐κB signaling pathway [20, 22]. An abnormality in the barrier correlates with a reduction in intercellular lipid species; the expression of peroxisome proliferator‐activated receptor α increases the synthesis of cholesterol and ceramides in keratinocytes. Quercetin can promote PPAR‐α activity, thereby inhibiting hyperkeratosis, parakeratosis, and acanthosis [28]. Meanwhile, quercetin can increase the expression of hyaluronan synthase 1 in epithelial cells, which can promote the accumulation of medium‐sized HA in keratinocytes [29]. Additionally, quercetin can be used to reconstruct the epidermis. Although topical HA is an effective way to strengthen the skin barrier, according to the results of this study, it is far less effective than topical CHSAG for improving the CEA and IGA scores and skin barrier function, and the topical CHSAG‐associated improvement in the skin barrier function is related to the improved CEA score.

The regulation of epidermal microbiota is closely related to the formation and repair of the skin barrier [30]. A complex microbiome is a hallmark of chronic nonhealing wounds, and pathogenic bacteria can evade the skin's immune system and reside within host cells. The overgrowth of demodexcan disrupt the skin microbiome and exacerbate inflammation in rosacea [31]. Demodex‐associated Bacillus proteins have been shown to induce abnormal wound‐healing responses in corneal epithelial cell lines [2]. In this experiment, while no significant difference in CEA scores was observed between Demodex‐positive and Demodex‐negative subgroups at baseline, the improvement in skin barrier function (hydration and TEWL) in the Demodex‐positive subgroup suggests that CHSAG may mitigate the skin barrier defects associated with Demodex overgrowth. This may contribute to the reduction in erythema, although other factors such as neurovascular dysregulation likely play a role. It has been shown that quercetin has high killing activity against demodex larvae, but its effect on the adult stage is unknown [32, 33]. The combination of quercetin and tetracyclines has a stronger antimicrobial effect [34]. During this experiment, compared with CG, EG not only reduced the CEA score of patients with epidermal Demodex infestation but also improved the skin barrier indicators more effectively, indicating that EG may promote the formation and repair of the skin barrier by inhibiting the imbalance in the abnormal Demodex on the skin.

Rosacea is a chronic disease that seriously affects the quality of life of patients. Traditional treatments are associated with limited efficacy, slow onset, long durations, high recurrence rates, and complex usage; furthermore, they often require simultaneous oral and topical administration of multiple drugs, thus causing psychological and economic burdens on patients. However, externally used CHSAG can effectively improve these shortcomings. Since quercetin blocks UV‐induced contact hypersensitivity, the 12 cases of adverse reactions with increased itching and burning sensation after 2 weeks in the CG may be related to the increased UV exposure with warmer temperatures during summer and seasonal transitions [35]. Simultaneously, a reduction in the incidence of pigmentation after inflammation and active anti‐oxidation and anti‐aging effects have been reported as additional benefits of externally used CHSAG for the treatment of rosacea, although the specific mechanism requires further investigation [36].

There are several limitations to this study; open‐label trials may introduce psychological expectations among participants, potentially affecting the results of the experiment. The single‐center design focusing only on the East Asian population, the small sample size, the short follow‐up time, and the possible interactions between systemic medications for topical medications may affect the observation of the long‐term efficacy and safety of CHSAG. Therefore, in future trials, studies without systemic therapy for more than 3 months in different geographic regions and ethnicities are necessary to observe the efficacy and safety of CHSAG. In conclusion, CHSAG effectively improved facial erythema, alleviated dryness and burning sensations, and improved patients' quality of life, although the optimal dosing for different populations remains to be determined through further research.

## Author Contributions

Z.X., B.Y., Y.Q., and S.Y. performed the research. B.X., B.Z., H.S., and N.W. designed the research study. Z.X. and J.W. contributed essential tools. Z.X. and J.W. analyzed the data. Z.X. and J.W. wrote the paper.

## Ethics Statement

The study was approved by the Institutional Review Board of the Second Affiliated Hospital of Xi'an Jiaotong University and met the requirements of the Helsinki Declaration.

## Consent

Informed consent was obtained from all subjects and/or their legal guardians for publication of identifying information/images in an online open‐access publication.

## Conflicts of Interest

The authors declare no conflicts of interest.

## Supporting information


**Figure S1.** Cohen’s d and Odds ratios for clinical symptom scores and representative images of patients.
Hydration (●) showed a progressive increase, reaching a strong effect size (> 0.8) at 12 weeks. TEWL (■) exhibited a negative trend, indicating enhanced skin barrier. RQoL score (▲) shows that EG’s RQoL score changed from slightly above CG to slightly below CG. Cohen’s d interpretation follows conventional thresholds: small (0.2), medium (0.5), and large (0.8) (a). Representative images of patients with rosacea after the external use of compound heparin sodium allantoin gel (CHSAG) or hyaluronic acid treatment at baseline and 8 weeks (c, d, e, f).

## Data Availability

The data that support the findings of this study are available from the Xi'an Jiaotong University but restrictions apply to the availability of these data, which were used under license for the current study and therefore are not publicly available. Data are however available from the first author upon reasonable request and with permission of the Xi'an Jiaotong University.
