# Supplementary figures and images for: Efficacy and Safety of Topical Compound Heparin Sodium Allantoin Gel (Main Components: Onion Extract Quercetin) for the Treatment of Rosacea
Source: J Cosmet Dermatol. 2025 Apr 3;24(4):e70129. doi: 10.1111/jocd.70129 (PMC11966348; doi:10.1111/jocd.70129)

a

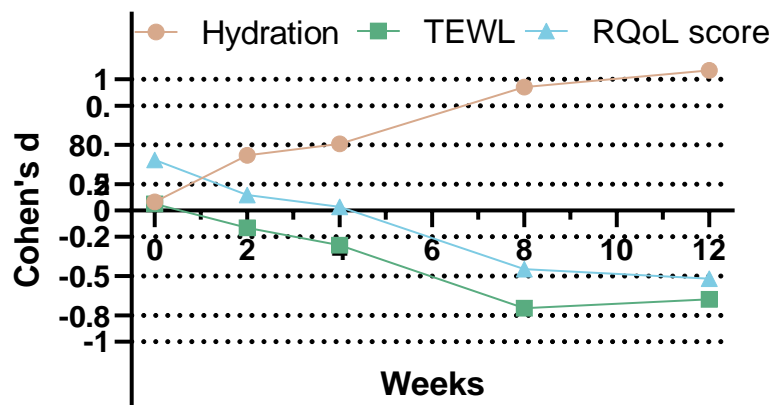

b

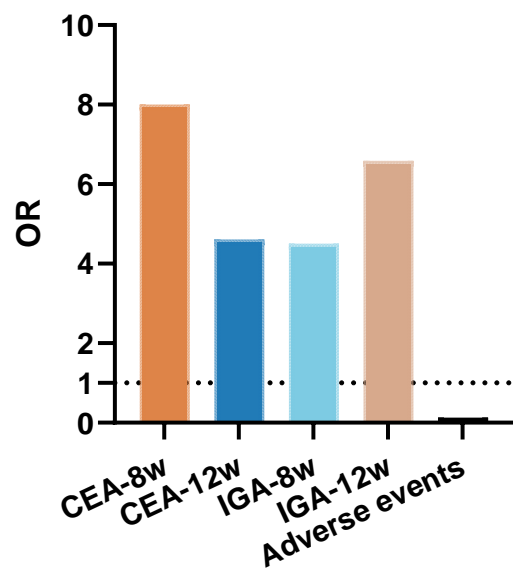

c

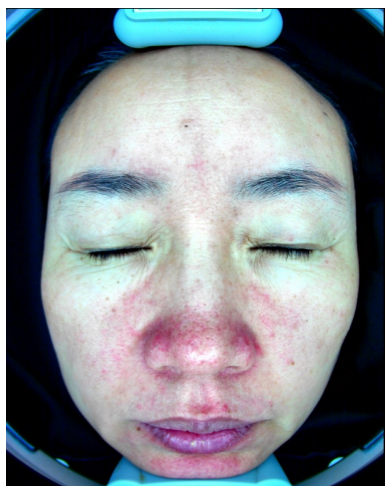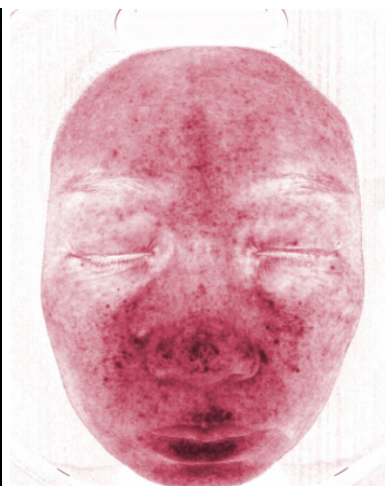

d

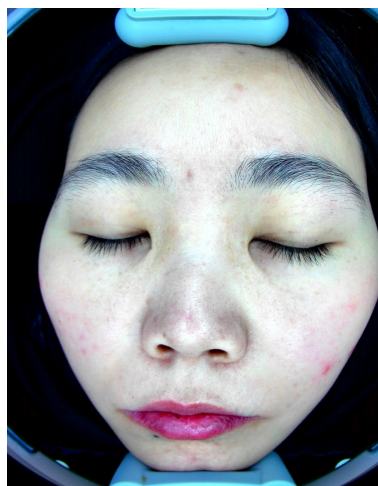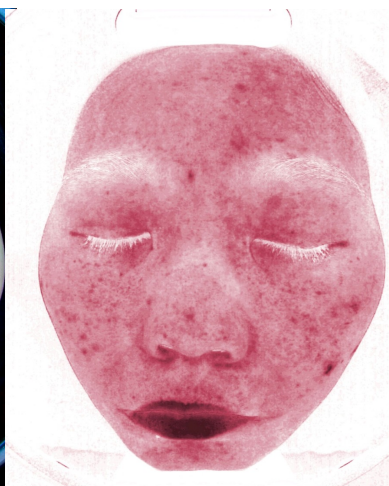

e

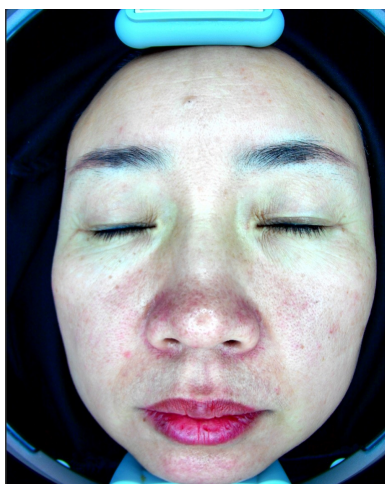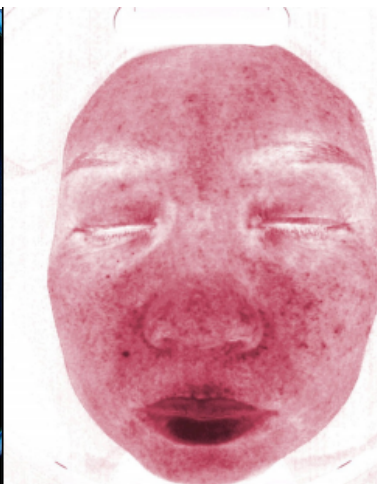

f

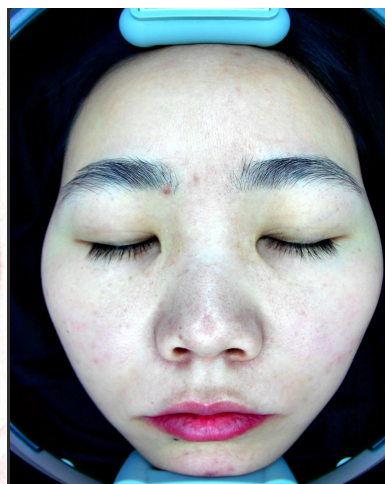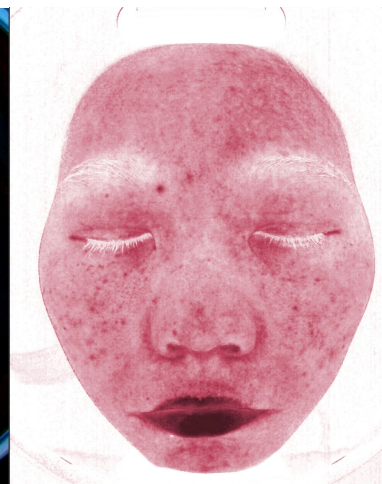

Supplement: Supplementary file 1 — Figure S1. Cohen’s d and Odds ratios for clinical symptom scores and representative images of patients. Hydration (●) showed a progressive increase, reaching a strong effect size (> 0.8) at 12 weeks. TEWL (■) exhibited a negative trend, indicating enhanced skin barrier. RQoL score (▲) shows that EG’s RQoL score changed from slightly above CG to slightly below CG. Cohen’s d interpretation follows conventional thresholds: small (0.2), medium (0.5), and large (0.8) (a). Representative images of patients with rosacea after the external use of compound heparin sodium allantoin gel (CHSAG) or hyaluronic acid treatment at baseline and 8 weeks (c, d, e, f). [file JOCD-24-e70129-s001.pdf]
